# Security Information and Event Management (SIEM): Analysis, Trends, and Usage in Critical Infrastructures

**DOI:** 10.3390/s21144759

**Published:** 2021-07-12

**Authors:** Gustavo González-Granadillo, Susana González-Zarzosa, Rodrigo Diaz

**Affiliations:** Cybersecurity Unit, Atos Research & Innovation, ATOS Spain, 28037 Madrid, Spain; susana.gzarzosa@atos.net (S.G.-Z.); rodrigo.diaz@atos.net (R.D.)

**Keywords:** evolution of SIEMs, SIEM enhancement, SIEM trends, critical infrastructures

## Abstract

Security Information and Event Management (SIEM) systems have been widely deployed as a powerful tool to prevent, detect, and react against cyber-attacks. SIEM solutions have evolved to become comprehensive systems that provide a wide visibility to identify areas of high risks and proactively focus on mitigation strategies aiming at reducing costs and time for incident response. Currently, SIEM systems and related solutions are slowly converging with big data analytics tools. We survey the most widely used SIEMs regarding their critical functionality and provide an analysis of external factors affecting the SIEM landscape in mid and long-term. A list of potential enhancements for the next generation of SIEMs is provided as part of the review of existing solutions as well as an analysis on their benefits and usage in critical infrastructures.

## 1. Introduction

Cybersecurity risks affecting industrial control systems (ICT) have grown enormously during the past couple of years, mainly due to increased activity by nation-states and cyber criminals. Attackers have become more sophisticated and dangerous and their appropriate and timely detection has become a real challenge. Examples of current cybersecurity incidents affecting IT and ICT are [1]: ransomware attacks; malware having impact on the utility’s ability to conduct business and operations; phishing campaigns directed to executives, executive assistants, SCADA engineers, IT administrators or other privileged users; business email compromise incidents, including account takeover or impersonation of executives; data leakage and thefts; social engineering to gather sensitive information from personnel.

According to a recent report from NIST [2], cybersecurity solutions in industrial control systems should provide real-time behavioral anomaly detection, enable faster incident management and allow for intelligent visualization of the network and all its interconnected nodes. Security Information and Event Management (SIEM) systems consider the aforementioned capabilities as built-in features.

In general, SIEMs have the capacity to collect, aggregate, store, and correlate events generated by a managed infrastructure [3]. They constitute the central platform of modern security operations centers as they gather events from multiple sensors (intrusion detection systems, anti-virus, firewalls, etc.), correlate these events, and deliver synthetic views of the alerts for threat handling and security reporting [4,5]. Besides these key capacities, there are many differences between the existing systems that normally reflect the different positions of SIEMs in the market.

Several companies have developed SIEM software products in order to detect network attacks and anomalies in an IT system infrastructure. Among them, we can find classical IT companies (e.g., HP, IBM, Intel, McAfee), others with more visionary options (e.g., AT&T Cybersecurity/AlienVault’s SIEMs), and promising tools to be taken into consideration in a SIEM context (e.g., Splunk).

In this paper, we review the most widely used security information and event management tools (commercial and open source) aiming at identifying their main characteristics, benefits, and limitations to detect and react against current attack scenarios. We provide an in-depth analysis of the features and capabilities of current SIEMs and focus on their limitations in order to propose potential enhancements to be integrated into current SIEM platforms. An analysis of external factors (e.g., political, economical, societal) that could potentially affect future SIEMs in the mid and long term is provided as a way to identify enablers and barriers to the new generation of SIEM systems. In addition, an overview of SIEM solutions in critical infrastructures is provided to identify potential usage of these tools. To the best of our knowledge, this paper is the first academic work to systematically analyze the current landscape of SIEM systems.

Paper Organization: The remainder of the paper is structured as follows: Section 2 introduces the main commercial and open source SIEM solutions available on the market. Section 4 analyzes the limitations of current SIEMs and presents potential capabilities for enhancements. Section 5 analyzes the future of SIEMs based on external factors. Section 6 proposes potential enhancements for the next generation of SIEMs. Section 7 provides an overview of the importance and usage of SIEM systems in critical infrastructures. Related works are presented in Section 8. Finally, conclusions are presented in Section 9.

## 2. SIEM Solutions

**Figure 1:**
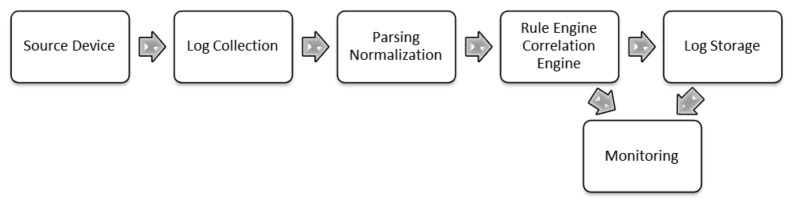
SIEM basic components.

Security Information and Event Management (SIEM) systems have been developed in response to help administrators to design security policies and manage events from different sources. Generally, a simple SIEM is composed of separate blocks (e.g., source device, log collection, parsing normalization, rule engine, log storage, event monitoring) that can work independently from each other, but without them all working together, the SIEM will not function properly [3]. Figure 1 depicts the basic components of a regular SIEM solution.

SIEM platforms provide real time analysis of security events generated by network devices and applications. In addition, even though the new generation of SIEMs provide response abilities to automate the process of selecting and deploying countermeasures, current response systems select and deploy security measures without performing a comprehensive impact analysis of attacks and response scenarios.

Besides these common features, current SIEMs present differences that classify them as leaders, challengers, niche players, or visionaries, according to the Gartner’s SIEM Magic Quadrant annual report. This section introduces the main SIEM solutions available on the market to date and provides the main advantages and drawbacks of each of them based on the most recent Gartner report and research works related to the SIEM technologies [6,7,8,9,10,11,12,13,14,15,16,17,18,19]. Please note that in this section we have considered the list of SIEM solutions proposed by Gartner during the last decade in their annual Magic Quadrant report, as such, the list of SIEM vendors presented in Table 1 makes reference only to the solutions selected and published by Gartner, and discards other commercial and open-source SIEMs that did not meet Gartner’s criteria.

### 2.1. SIEM Classification

The analysis and evaluation of security systems have been widely proposed in the literature. While some research focuses on the commercial aspects, others concentrate on the technical features that could be improved in current SIEM solutions. Well known institutions like Gartner [20], for instance, propose a commercial analysis of SIEM systems based on the market and major vendors, for which a report is released on an annual basis to position SIEM vendors as market leaders, challengers, niche players, or visionaries.

Other security institutions (e.g., Techtarget (http://searchsecurity.techtarget.com/ accessed on 12 January 2021) and Info-Tech Research Group (http://www.infotech.com/ accessed on 12 January 2021)), have widely reported on the capabilities of SIEM solutions and on the way SIEM vendors can be compared and assessed. Techtarget, on the one hand, releases periodic electronic guides about securing SIEM systems and how to define SIEM strategy, management and success in the enterprise [21]. Info-Tech, on the other hand, provides technical reports on the SIEM vendor landscape [22] focusing on the benefits and drawbacks of major commercial SIEMs. Both organizations take the Gartner Magic Quadrant as the baseline for their analysis.

During the last decade, Gartner has classified SIEM solutions as leaders (organizations that execute well against their current vision and are well positioned for tomorrow), visionaries (organizations that understand where the market is going or have a vision for changing market rules, but do not yet execute well), niche players (organizations that focus successfully on a small segment or are unfocused and do not out-innovate or outperform others), and challengers (organizations that execute well today or may dominate a large segment, but do not demonstrate an understanding of market direction).

Table 1 shows the evolution of SIEM solutions (and SIEM vendors) from 2010 to 2020. Note that the latest report to date was released in January 2020 (no report was delivered in 2019). From Table 1, the star indicates those that have been leading the market, challengers are identified with a lozenge, niche players are identified with a triangle, and visionaries are identified with a square. It is important to highlight that very few of them appeared every year in the top ranking assessment during the whole decade period. This is the case of RSA, the security division of EMC Corporation (Dell Technologies), which offers a NetWitness Platform evolved SIEM; IBM, which offers a tool called Qradar; NetIQ/Microfocus/ArcSight, offering the ArcSight Enterprise Security Manager; McAfee/Intel, offering the McAfee Enterprise Security Manager, and LogRhythm, offering the Nextgen SIEM platform.

Note that some solutions have been merged to keep with the changes and evolution of the market. This is the case of IBM and Q1 labs (that offered a joined solution in 2012 and 2013); NetIQ and Novell (2012); HP and ArcSight (2013); AccelOps and Fortinet (2016), and more recently, Micro Focus and ArcSight, as well as Micro Focus and NetIQ (2017). Some of these joined solutions are no longer available in 2021.Another important aspect to note is that some SIEM vendors have been in the top Gartner classification list ever since they first appeared in the market (e.g., Splunk, AlientVault/AT&T Cybersecurity, SolarWinds, EventTracker, Fortinet, MicroFocus). Some others have joined the list in the past couple of years with User and Entity Behavior Analytics (UEBA) features (i.e., Manage Engine, Venustech, Rapid7, Exabeam, Secureonix, LogPoint, and HanSight).

A recent study [22] considers 22 players in the 2020 SIEM vendor map based on three main capabilities: (i) threat intelligence detection, (ii) compliance, and (iii) log management. Besides threat intelligence, compliance, and log management, SIEM developers are considering UEBA capabilities and smart dashboards as innovations to be added to their solutions. As a result, new SIEM systems will help security administrators with pre-built dashboards, reports, incident response workflows, advanced analytics, correlation searches, and security indicators [23]. In addition, an in-depth analysis of SIEMs extensibility [19] revealed that current SIEM solutions need to improve features such as behavioral analysis, risk analysis and deployment, visualization, data storage, and reaction capabilities, in order to keep up with the market.

### 2.2. SIEM Tools

Considering the previous information about SIEMs, Table 2 summarizes some of the most promising SIEMs to date.

## 3. SIEM Features and Capabilities

Fundamentally, all SIEMs have the capacity to collect, store, and correlate events generated by a managed infrastructure [64]. Besides these key capacities, there are many differences between existing systems that normally reflect the different positions of SIEMs in the market. This section provides a list of features to be considered in the analysis of SIEM solutions. Based on our experience with different commercial SIEMs and contrasting the identified information related to the usage of commercial and open-source SIEMs from the literature, Table 3 summarizes this analysis and assesses each SIEM feature as low/basic (poorly implemented or not implemented at all), average (partly implemented), or high/advanced (fully implemented) for the most promising SIEM solutions described in Table 2. Please note that this assessment only includes the basic configuration of the selected SIEM solutions, no additional features (add-ons) are considered in the analysis.

Correlation rules: The success of detecting an event by a SIEM relies on the power of the correlation rules. While most SIEMs possess basic correlation rules, few of them have robust search capabilities and support search processing languages to write complex searches that can be used on the SIEM’s data.

Data sources: One of the key features of a SIEM system is the capacity for collecting events from multiple and diverse data sources in the managed infrastructure. Most SIEMs support several types of data sources natively, including both the supported sensors, and the supported data types (e.g., threat intelligence). For other solutions (e.g., QRadar, USM) such a feature could be supported by additional components integrated to the SIEM. This feature evaluates the natively supported data sources and the possibility for a SIEM to automatically customize them.

Real time processing: This feature considers the ability of a SIEM to handle real-time data under constant change. It evaluates the real-time controls, monitoring, and pipelining capabilities deployed by the tool in preventing or reacting to cybersecurity incidents, as well as the performance computation capabilities that SIEMs have to analyze millions of events in real time. All the studied SIEMs have advanced real time processing capabilities.

Data volume: Analyzing large volumes of data coming from different sources is important to gain more insights from the collected events and to have a better monitoring. However, keeping large volumes of collected data in a live SIEM system is often costly and impractical. This feature evaluates the possibility of current systems to support large volumes of data for correlation, indexing and storage operations.

Visualization: One of the key factors that hinder the analysis of security events is the lack of support for proper data visualization methods and the little support provided for interactive exploration of the collected data. It is therefore important to understand the capabilities of the analyzed systems in terms of creation of new data visualization methods and custom dashboards.

Data analytics: More recent versions of leading SIEMs support extensive integration with application and user-based anomaly detectors. These capabilities include the analysis of the behavior of employees, third-party contractors, and other collaborators of the organization. For this, the SIEM must comprise the management of user/application profiles and the use of machine learning techniques for detecting misbehavior.

Performance: This feature evaluates the performance of a SIEM solution in terms of computational capacity, data storage capabilities (e.g., read/write), rule correlation processing (e.g., high performance correlation engine), as well as data search, index, and monitoring.

Forensics: In addition to logging capabilities, some SIEMs (e.g., ArcSight, LogRhythm) offer built-in network forensic capabilities that include full session packet captures from network connections considered as malicious aiming at converting packet data into documents, web pages, voice over IP, and other recognizable files. Some other products (e.g., QRadar, Splunk) are able to save individual packets of interest when prompted by a security analyst, but do not automatically save network sessions of interest [16], and the rest of studied solutions have no built-in network forensic capabilities.

Complexity: SIEMs are known for being difficult to deploy and manage. However, it is important to understand if the analyzed system can be installed for testing with low or moderate effort. From the eight studied SIEMs, ArcSight is the tool with the highest complexity for deployment and management, whereas LogRhythm and Splunk are seen as easy and friendly tools to install, deploy, and use.

Scalability: This feature considers the ability for a SIEM deployment to grow not only in terms of hardware, but also in terms of the number of security events collected at the edge of the SIEM infrastructure. The new digital transformation leads to more sensors and more devices (e.g., servers, agents, nodes) connected to the same network.

Risk analysis: Recent versions of leading SIEM systems (e.g., QRadar, LogRhythm, Splunk) include features for doing risk analysis on the assets of the managed infrastructure. This feature evaluates if the SIEM natively supports risk analysis or if it can be integrated with external appliances for that purpose.

Storage: Considering that SIEMs generally store information for no more than 90 days, this feature evaluates the length at which current SIEM technologies keep data stored in their systems for further processing and forensics operations.

Price: This feature evaluates the licensing method associated to the SIEM solution (e.g., enterprise, free, beta, premium) and the limits in the number of users, queries, index volumes, alerts, correlations, reports, dashboards, and automated remedial actions. Most of the studied solutions are very expensive, except for LogRhythm, USM, and SolarWinds, with more reasonable costs and the possibility to use open source solutions with more limited capabilities.

Resilience: Resilience or fault tolerance is an important feature of any critical monitoring system. It is important to understand what the fault tolerance capabilities of existing SIEMs are, for example, if the correlation engine supports fault tolerance; the way disaster recovery and replication are supported on the event storage; if the connectors support high availability features.

Reaction and reporting capabilities: This feature studies the actions that are natively supported by the SIEM to react against security incidents (including sharing and reporting capabilities) and the way such actions are expressed to the correlation engine.

UEBA: This feature evaluates if the SIEM solution presents native User and Entity Behavior Analytics (UEBA) capability, or if it provides integration with third-party UEBA solutions.

Security: This feature evaluates the ability to implement security automation as well as native encryption capabilities present in the SIEM during the monitoring, detection, correlation, analysis, and presentation of the results.

## 4. Limitations of Current SIEMs

Even though the new generation of SIEMs provides powerful features in terms of correlation, storage, visualization, and performance, as well as the ability to automate the reaction process by selecting and deploying countermeasures [65,66], current response systems are very limited and countermeasures are selected and deployed without performing a comprehensive impact analysis of attacks and response scenarios [67].

In addition, most SIEMs support the integration of new connectors or parsers to collect events or data, and provide APIs or RESTful interfaces to collect the events at a later date. These mechanisms allow creating add-ons and extensions to existing systems. Future SIEMs must exploit this feature in order to enhance the quality of the events fed to the system (e.g., using new monitoring systems or collecting external data from open source intelligence) through custom connectors, and provide new visualization tools by collecting data from the SIEM data repository.

This section details the main limitations found in current SIEM solutions and provides some perspectives on possible enhancements.

### 4.1. Incomplete Data

Although current SIEMs deal with tons of data, none of them have all the data needed to process and detect all security incidents. The reason is that it is not cost-effective to capture and process all the required data. Typically, all SIEMs correlate logs from VPNs, firewalls, domain controls, failed connections, etc. Most SIEMs are able to correlate logons, malware, and web logs, but just few of the current SIEMs correlate DNS traffic, end-point data logs, and email logs. As a result, it is not possible to know who everyone is in the system [68].

Identity is fragmented, people possess shared accounts and different roles are associated to the same user, but by law, we cannot disclose the identity of a given person since it generates privacy issues, as presented in the General Data Protection Regulation (GDPR) [69]. If the SIEM is not able to capture all data about users and high value assets, correlation will never work properly, resulting in large number of false positives and negatives. The next generation of SIEMs must, therefore, meet the privacy requirements of the GDPR while providing enough information for analysts to identify security incidents [70].

The reviewing of existing SIEMs allowed us to confirm that these systems do not provide high-level security risk metrics. A major advance on current SIEMs will be the development of useful operational metrics that allow SOCs to make decisions supported by quantitative evidence, where uncertainty in the measures is explicitly stated, and with better visualization support to enable better communication of these decisions to the relevant stakeholders in the organization [71,72]. Such measurements must be supported on several layers of defense (e.g., firewalls, IDSs, anti-virus products, operating systems, applications) and different products of each type.

Although cost sensitive metrics are hard to compute due to the difficulty in estimating security costs of organizations, novel SIEMs must approach this category of metrics using high-granularity estimation of costs.

Future SIEMs must explore and implement novel unsupervised techniques that combine statistical and multi-criteria decision analysis to automatically model applications and users’ behaviors, and subsequently identify anomalies and deviations from known good behaviors that are statistically relevant. This will lead to the deployment of enhanced application monitoring sensors, which will feed SIEM systems with diverse types of events that can be correlated with more traditional security events collected from host and network-based appliances.

By combining anomaly-based events with those provided by more traditional heuristic and signature based tools, SIEMs will improve the false positive rates of these components, which have traditionally been the main stumbling block of their wide adoption in real operations.

### 4.2. Basic Correlation Rules

SIEM platforms provide real-time analysis of security events generated by network devices and applications [3]. These systems acquire high volumes of information from heterogeneous sources and process them on the fly. Their deployment thus focuses, firstly, on writing ad hoc collectors and translators to acquire information and normalize it, and secondly, on writing correlation rules to aggregate the information and reduce the amount of data. This operational focus leads SIEM implementers to prioritize syntax over semantics, and to use correlation languages that are poor in terms of features [73]. However, as the number of attacks, and thus the diversity of alerts received by SIEMs increases, the need for appropriate treatment of these alerts has become essential.

Current SIEM correlation rules are weak [74]. Most of them use basic boolean chaining of events that check for a specific attack path (one from the many thousands of possibilities). Very few SIEM solutions have a built-in advanced correlation engine able to perform the deviation and historical correlation useful for instance to check after zero-day attack detection.

### 4.3. Basic Storage Capabilities

For most existing SIEM solutions, once data is archived and is out of the live system, the SIEM will not use it. Moreover, how archived data is handled or where it is stored or transferred is up to the user and is usually done manually. As there are diverse options for where to store archived data some SIEM users will opt for attached storage, others will use an in-house distributed file system, e.g., Hadoop Distributed File System (HDFS) [75], a commercial cloud storage solution like Amazon S3, Amazon Glacier, or even use “scp” operations to another device.

Regardless of the archiving solution employed, the actual archiving process consists of running scripts that are often custom built for a specific IT environment. Therefore, a script used by one customer may not be useful for another customer’s need, and a change in the archive option requires rewriting the archiving script.

Furthermore, archiving retired data from a SIEM can be costly and can pose security and reliability problems if the archived data is not handled correctly.

Current infrastructures usually store raw events for a limited amount of time (e.g., 6 months) to limit the storage space used for such archival (e.g., 6 TB). Given that some advanced persistent threats are detected many months after their inception in the system [76], such storage capabilities might be insufficient to help with certain incidents.

Although promising, most companies avoid using the cloud due to concerns related to the confidentiality of the events (that contain sensitive information [69]) and concerns related to trusting such important data to third parties [77].

The goal of future SIEMs must focus on offering a secure and elastic solution for data archival regardless of the data retention needs with the ability to customize policies to fit retention requirements.

### 4.4. Reliance on Humans

Research in SIEM technologies has traditionally focused on providing a comprehensive interpretation of threats, in particular to evaluate their importance and prioritize responses accordingly. However, in many cases, threat responses still require humans to carry out the analysis and make decisions with respect to understanding the threats, defining the appropriate countermeasures, and deploying them. This is a slow and costly process, requiring a high level of expertise, and remains error prone nonetheless. Thus, recent research in SIEM technologies has focused on the ability to automate the process of selecting and deploying countermeasures.

According to Scarfone [78] automated reactions must consider: (i) time-line: the time that a SIEM takes to detect an attack and direct the appropriate security control to mitigate it; (ii) security: the communications between the SIEM and the other security controls protected so as to prevent eavesdropping and alteration; (iii) effectiveness: the ability for a SIEM product to stop attacks before damage occurs.

### 4.5. Basic Reaction and Reporting Capabilities

Traditionally, SIEMs support the creation of security directives for detecting suspect behavior in the system and reporting alarms. However, these directives/rules could, in principle, be used to trigger actions for modifying the managed infrastructure (e.g., changing the configuration of firewalls or NIDS).

For some SIEMs, it is possible to use automatic triggers to perform external actions (e.g., send emails, execute scripts, open tickets), usually through a command line. However, most of these systems do not provide pre-configured and customized actions to be triggered when a specific condition or set of conditions are fulfilled. They generally focus on the creation, distribution, and management of reports.

In addition, some SIEMs require the use of additional solutions (e.g., add-ons, appliances, extensions) to provide automatic reactions when an alarm is detected.

An important part of the design for security is defense in depth [79] using layers of defense that reduce the probability of a successful attack (or at least contain its effects). This requires the use of diversity, including but not limited to the use of multiple intrusion detection systems (IDSs) and disparate open-source intelligence data (e.g., infrastructure-related information about security from open-source intelligence data available on diverse sources from the internet). There has been only sparse research on how to choose among alternative layered defenses; occasionally, unsuitable models appear relying on the naive assumption of independent failures between the diverse components [80]. Security engineers have little or no theory to guide their decisions about diversity, although unaided intuition can be very misleading (e.g., Littlewood and Wright [81]).

SIEMs already provide the functionality for reading logs from multiple different security monitors and detection tools at different layers. Future SIEMs should build tools that allow consolidation of outputs from multiple diverse monitors of similar type, which may be monitoring similar types of assets. This will help in improving the accuracy of the detection, and reducing the false alarm rates that are reported back to the SOCs.

Even though the need and relevance for providers of security services having Cyber Security Reporting Systems (CSRS) was identified almost two decades ago [82], there is still a lack of solutions focused on the management and generation of mandatory incident reporting according to different regulatory frameworks. In addition, although the growing quantity of existing regulations and legislation addressing cybersecurity incidents has created a need for studies on cybersecurity incident reporting for specific areas (e.g., nuclear facilities [83], safety-critical ystems [84]), currently this functionality is very limited in most commercial and open-source SIEMs. Solutions such as IBM QRadar, AT&T USM anywhere, or Splunk generate reports about detected security incidents, nonetheless, such reports do not follow standards or common templates, and the information included does not cover what is required for mandatory incident reporting to the different supervisory authorities [85].

### 4.6. Limited Data Visualization

During the reviewing of the state-of-the-art of existing SIEMs, we observed that the reporting and data visualization capabilities are limited in terms of supporting the effective extraction of actionable insights from the huge amount of data being collected by the systems. Although all SIEMs offer data visualization capacities to their users, most often the visual representations are generic, not designed with particular user needs in mind, or even are too highly rudimentary to have any significant effect on how the generated data is utilized [86].

In addition, existing systems do not have the capacity to use diverse data modes, e.g., statistical modeling outputs, OSINT data collections, or comprehensive models of user behavior. These novel data facets, when combined with the data already being gathered, offer challenges and opportunities for a new generation of SIEMs.

To enhance the visualization capability of existing systems, SIEMs must focus on flexible platforms able to work with several data sources that carry heterogeneous characteristics and with data that is under constant change, i.e., real-time streaming data. In addition, visualization must enable security analysts to better profile the system with novel representations that communicate the provenance of an attack, ongoing activities, vulnerabilities, and the characterization of sessions/users [87,88].

## 5. The Future of SIEMs

The changing nature of security threats, the proliferation of mobile devices, globalization, the explosion of social media, and quick changes in regulation are speeding the evolution of Security Information and Event Management. The purpose of this section is to analyze the external factors that could potentially affect the future of SIEM systems and their related technologies in the mid-term and long-term based on political, economical, societal, technological, legal, and environmental factors [89,90,91]. We employ the PESTLE [92] analysis aiming at identifying the enablers and/or barriers that could directly or indirectly affect the evolution of SIEMs.

### 5.1. Political Factors

Protection of individual properties and sensitive business or personal information in the cyberspace is becoming critical and political organizations must take part in this. They must design the security framework, principles, and rules to reduce the risks in the population. This risk may economically affect private companies and public institutions. These regulations may affect the evolution of SIEMs in the future, since, in some instance, they analyze sensitive information to detect security events in the network.

Recently the EU Commission announced an increase (expected to trigger EUR 1.8 billion of investment by 2020) in the investment on cybersecurity in order to put more efforts to reduce cyber-threats in the European Union [93]. In addition, according to Andrus Ansip, Vice-President for the Digital Single Market, without trust and security, there can be no Digital Single Market. Europe is proposing concrete measures to strengthen resilience against cyber-attacks and secure the capacity needed for building and expanding the digital economy. Furthermore, Gunther H. Oettinger, Commissioner for the Digital Economy and Society, considers that Europe needs high quality, affordable, and interoperable cybersecurity products and services [93].

This is an initiative of the Commission to establish contractual Public Private Partnership [94] (cPPP) on cybersecurity between the European Union and the European Cybersecurity Organization. The adoption and evolution of SIEMs can then be empowered by this investment in cybersecurity.

### 5.2. Economic Factors

Among the economic factors that will affect the future of SIEMs the following can be highlighted:

- Short term/temporary work. In 2014 the main type of employment relationship in the EU was full-time permanent contracts, with 59% of the share of employment, although this is decreasing while the share of non-standard forms of work is increasing. If this trend continues, it may well become the case that standard contracts will only apply to a minority of workers within the next decade [95]. Due to the new types of work, tending to shorter term jobs, people do not stay in the same company for a long time, especially in the first period of their career. The consequence is that companies need to minimize the employee’s ramp up to learn a new tool, or a new way of working. Therefore, this factor makes it essential that future SIEMs have improved and more friendly interfaces at the level of decision taking, configuration rules, links to new sources, and sensors.
- Freelance. Self-employment is increasing against the usual company paid employment [96]. Freelancers do not work for a company as an employee but as a service provider. This type of work may be a threat for companies because the devices used by freelancers do not belong to the IT department and cannot be easily monitored. Furthermore, they do not have strong bonds with the company that hires their services. However, freelance cybersecurity consultants can be a good choice for SIEM providers because they may possess a wider knowledge about potential threats affecting an organization, since they accumulate a lot of experience from different companies.
- Cyber security jobs are continuously growing. The estimated growth in cybersecurity jobs is of 35% by 2020 [97]. This reflects the importance of cybersecurity for the companies, and that can be an opportunity for SIEMs to grow in the market.
- Bigger companies, globalization. The global market makes it easier for big technological companies to survive and grow more [98]. However, the level of criticality of that information may be higher. Future SIEMs should be dimensioned for such big companies and global networks.
- Small and medium sized enterprises. SMEs will become bigger targets of cyber-attacks in the future [99]. They should be the new target for SIEM market growth, making models like SIEM as a service more attractive to SMEs.

### 5.3. Societal Factors

Society is becoming strongly dependent on information and communications technology (ICT), which is leading to a rapid social, economic, and governmental development. The following introduces how the changes in societal habits related to technology will affect the future of SIEMs.

- Generation Z. Modern generations understand the world as a big network in which everything is connected to the internet. It can be assumed that people of the future will be more aware of cybersecurity and will bring companies clearer awareness of the risks associated to threats in the network [100].
- Growth of social networks. There is a huge growth of social networks usage among the young generations in the last few years. Social network activity is a source of data that should not be disregarded, and it can be of very high importance in security events analysis [101].
- Cyber-attacks. In the new connected societies, the development of the internet has led to a new type of attacks, i.e., cyber-attacks. Attacks to critical infrastructures can be considered the new weapons, which makes SIEMs essential in any infrastructure in which data is of relevance or whose attack may cause operation disruption, even damage to population, not only from a single company’s perspective but also from users, citizens, and (more generally) people’s perspective [102].
- Deep web. The deep web is the part of the World Wide Web whose contents are not indexed by standard search engines [103]. This can be considered as a barrier by SIEM systems, since it makes it difficult to retrieve data from the network.

### 5.4. Technological Factors

Among the technological trends that will affect the way SIEMs evolve in the future, the following can be highlighted:

- Cloud storage. This technology can be clearly seen as an enabler in SIEM technology since big data analytics of network events can be performed in a more efficient way, without worries about the amount of logs, information, etc., that are stored.
- Cloud service integration. This is treated separately to cloud storage because it is more focused on executing software in a remote server, and not only keeping data “statically” in a cloud infrastructure. This technology makes it possible to ensure scalability and high availability of software applications since they are not restricted to the hardware of a local server, and can be launched from anywhere.
- Mobile technologies. The growth of mobile devices brings new threats that should be analyzed by SIEM systems. In this respect, it is a trend that employees use company-owned devices as well as personal devices for office work. A need would be to secure corporate data. Working at home, e.g., with a personal computer, what now is commonly called BYOD (Bring Your Own Device), is a trend in cybersecurity [104]. However, this leads to several potential problems: BYOD devices are not managed by the IT team so they are not under the policy control of the company; some BYODs do not have any security solution pre-installed; data in these devices is not encrypted; applications installed in those devices cannot be tracked.
- Big data analytics. As introduced before, SIEMs are evolving to data analytics systems. Data in a connected environment grows exponentially and makes it necessary to have powerful analysis tools capable of real time analysis of events, support to decision making, etc. The growth in data analytics methods is clearly an enabler for SIEM systems.
- Machine learning technologies. New high performance computers, with powerful hardware and modern programmatic languages, together with the data analytics explained above, are making it possible to create data models fed by the experience of cause-effect analysis. SIEMs can take advantage of these technologies to make event detection and decision making smarter [105].
- Internet of Everything. The Internet of Everything (IoE) [106] is a ubiquitous communication network that effectively captures, manages and leverages data from billions of real-life objects and physical activities. It extends the concept of Internet of Things (IoT) by also including people, processes, locations, and more. The impact of this technology on SIEMs is that they provide large amount of data and events for analysis.
- 5G Networks. 5G represents the next generation of communication networks and services, an approach for fulfilling the requirements of future applications and scenarios. This technology will increase the data transfer speed, and then could affect the amount of data analyzed by a SIEM in a network per time unit. This can impose a difficulty for SIEMs in events detection.
- Social media analytics. Social networks like Twitter provide a wealth of information that may be explored by cybersecurity companies as well as by hackers, as attack victims use on-line social media to discuss their experience and knowledge about attacks, vulnerabilities, and exploits.

### 5.5. Legal Factors

In January 2012, the European Commission proposed a comprehensive reform of data protection rules in the EU. On 4 May 2016, the official texts of the Regulation and the Directive REGULATION (EU) 2016/679 were published in the EU Official Journal [107]. While the Regulation entered into force on 24 May 2016, it was set to apply from 25 May 2018. The EU Member States had to transpose the directive into their national law by 6 May 2018.

The objective of this new set of rules is to give back citizens the control over their personal data, and to simplify the regulatory environment for business. The data protection reform is a key enabler of the Digital Single Market which the Commission has prioritized. The reform will allow European citizens and businesses to fully benefit from the digital economy [108].

A number of provisions of the Directive contain a substantial degree of flexibility in order to find an appropriate balance between protections of the data subject’s rights on the one side and on the other side the legitimate interests of data controllers [109].

In order to understand how this regulation may affect the data collected by SIEMs, we can see for example how EC understands the propriety of the IP address in a network (commonly analyzed by security software). In the internet, every computer is identified by a single numerical IP address of the form A.B.C.D. where A, B, C, and D are numbers in the range of 0 to 255. The working party has considered IP addresses as data relating to an identifiable person, especially in those cases where the processing of IP addresses is carried out with the purpose of identifying the users of the computer (for instance, by copyright holders in order to prosecute computer users for violation of intellectual property rights), the controller anticipates that the “means likely reasonably to be used” to identify the persons will be available, for example, through the courts appealed to (otherwise the collection of the information makes no sense), and therefore the information should be considered as personal data [110].

Consequently, the way SIEMs process and store data must be in line with the directives on data protection. Moreover, the regulation in data protection affects the SIEMs in the way they can store the data, where the database is located, and that the stored data is kept with adequate level of protection.

### 5.6. Environmental Factors

SIEM challenges will continue to evolve as security managers grapple with cloud services, mobile, the Internet of Things, and other new technologies the IT department does not always control. IoT will be a huge factor as it drives the number of endpoints vulnerable to attackers [111,112]. It gets harder for cybercriminals to infiltrate computers but is still fairly easy to hack cameras, refrigerators, microwaves, Bluetooth tools, and other connected devices and use them as an attack vector.

The growth of cloud, especially for small and medium businesses (SMBs), has transformed how businesses store and handle data. Companies once intimidated by the high price of data storage, benefit from SIEM providers like ArcSight, Nitro, and others that deploy modules from the cloud [111].

Cloud services and IoT devices will rapidly generate increasing amounts of data, and SIEM systems will have to adapt by learning to collect and organize the influx of information.

## 6. Potential Enhancements of Future SIEMs

SIEMs are mostly used in IT infrastructures where automated detection and reaction is possible. However, in critical infrastructures, these tools require manual intervention and in-depth analysis of events before implementing a security countermeasure. This section provides potential enhancements on the future generation of SIEMs considering the following aspects:

### 6.1. Diverse Security

Enhancing SIEMS with diversity-related technologies provides a major improvement of current solutions. Special attention must be paid to diversity measures—i.e., how similar or different security protection systems, vulnerabilities, attacks, etc., are from each other. These types of diversity metrics are less studied in the literature compared with metrics for individual components.

Future SIEMs must define security metrics that consider quantitative and probabilistic methods to support decisions on how best to combine multiple defenses given a threat environment [113,114]. This involves understanding how the strengths and weaknesses of diverse defenses add up to the total strength of the system.

The security community is aware of diversity as potentially valuable [115]. The literature touches on the use of ensemble methods to assess the results of classification systems for security [116]; however, SIEMs should focus in diverse inputs rather than the aggregation of diverse machine learning techniques.

### 6.2. OSINT Data Fusion

A potential enhancement for current SIEMs could be the use of language processing to identify threats from the use of keywords that typically indicate a threat in major languages; such as “ddos”, “security breach”, “leak”, and more [117,118,119]. This information can be used to tag OSINT data as relevant or irrelevant. In addition to the type of threat, other information from the OSINT sources such as location and entities involved could also be extracted to provide a more comprehensive description of the threat. The prediction confidence of the classifier can be included in the data sent to SIEMs, which will help to avoid the issue of false alarms.

### 6.3. Enhanced Visualisation

To enhance the visualization capability of existing SIEMs, we identify the following improvements [86]:

- Design and develop a rich set of specialized visualization models that handle diverse types of data e.g., high-dimensional, temporal, textual, relational, spatial.
- Provide effective overviews, interactive capabilities to focus on details, and mechanisms to compare individual and/or groups of data instances.
- Design and develop visualization models capable of handling the dynamic nature of the data (e.g., streaming system activity logs, OSINT data, etc.) to support real-time analysis and decision-making.
- Develop a visual summary of user activities that reveals common/abnormal patterns in a large set of user sessions, compares multiple sessions of interest, and investigates in depth of individual sessions.

### 6.4. Enhanced Storage

In addition, archiving retired data from a SIEM can be costly and can pose security and reliability problems if archived data is not handled correctly. A potential solution for these issues could be to develop a SIEM extension that handles data archiving in a reliable, flexible, and secure manner leveraging public Clouds (e.g., Amazon S3, Amazon Glacier, Windows Azure, Blob Store, etc.). The goal is to offer a secure and elastic solution for SIEM data archival regardless of the data retention needs with the ability to customize policies to fit retention requirements [120].

### 6.5. Integration with Security Orchestration Automation and Response (SOAR)

SOAR refers to three main security topics: (i) security orchestration, focusing on the workflow management, integration and unification of components involved in security operations; (ii) security automation, responsible for automating repetitive controls, tasks and processes taking place in security operations; (iii) security incident response, focusing on the identification and management of security threats and incidents. SOAR solutions would ideally complement the capabilities of current SIEMs, which together with additional technologies such as Threat Intelligent Platforms (TIPs) [121], Endpoint Detection and Response (EDR) [122], or Next-Generation Firewalls (NGFW) [123] are seen as a proactive platform for early detection, prevention, and response of cybersecurity threats and attacks [124,125,126].

The next generation of SIEMs must integrate evolved and adaptive SOAR solutions with advanced capabilities that enable dynamic interactions at all phases of the incident workflow to quickly deal with existing and emerging threats [127,128]. Examples of enriched adaptive SOAR include the NextGuard Adaptive security Operations suite from Nokia NextGuard (https://www.nokia.com/networks/solutions/netguard-adaptive-security-operations/ accessed on 7 June 2021), the Splunk adaptive Operations Framework (AOF) (https://www.splunk.com/en_us/solutions/solution-areas/security-and-fraud/adaptive-response-initiative.html accessed on 7 June 2021), and the Integrated Adaptive Cyber Defense (IACD) (https://www.iacdautomate.org/ accessed on 27 May 2021).

### 6.6. AI/ML Capabilities

In order to improve detection, correlation and reaction capabilities, the next generation of SIEMs should integrate AI/ML technologies in their core engines [129]. AI technologies in SIEMs offer predictive capabilities particularly useful for the analysis of abnormal behavior of network traffic, tools, and users. Few of the current SIEM solutions (e.g., LogRhythm NextGen SIEM Platform (https://logrhythm.com/products/features/ai-engine/ accessed on 28 May 2021), QRadar SIEM (https://www.midlandinfosys.com/ibm-power/all-categories/ai-security-siem-qradar-uba.html accessed on 28 May 2021)) use machine learning (ML) to learn about threats as they acquire threat intelligence and deflect attacks in the filed [130,131].

One step forward for cyber threat detection, mitigation, and prevention is to consider AI/ML in SOAR solutions which would be ideally integrated in SIEM platforms. AI/ML-powered defense systems are able to analyze large amount of data and identify suspicious patterns in real-time (or near real-time). The main targets for AI/ML applications include intrusion detection (network-based attacks), phishing and spam (emails), threat detection and characterization, and user behavioral analytics [132].

AI-based SIEMs are able to make decisions and/or change their behavior accordingly, which improves detection capabilities by discovering more blind spots, reduces dependencies of manual intervention, as some reactions can be automated, and minimizes false positive rates, as algorithms have the ability to accurately classify data as normal or abnormal. Ideally, next-generation SIEMs should combine rule-based analysis with the one provided by AI technologies to detect users deviations, identify changes in users activity vs. frequency, detect anomalous deviations from peer groups, prioritize users and assets, and respond to threats quickly and accurately [133].

Improvements of future SIEMs should also include creating sensors that rely on unsupervised statistical learning approaches to firstly create a baseline for normal entity behavior (users and applications alike). The scope is to be able to highlight anomalies and/or deviations from this pattern by using a SIEM scoring-alerting system. In terms of User Behavior Analysis (UBA), a set outlier detectors or classifiers as well as other unsupervised machine learning algorithms could be used in order to manage user/application profile [134,135,136].

### 6.7. Other Potential Enhancements

The review of existing SIEMs revealed that these systems do not provide high-level security risk metrics. The next generation of SIEMs must pursue the development of risk-based metrics considering several layers of dependencies such as hosts, applications, middleware, and services. These will allow scoring the risk for the different operational and functional areas. Attack propagation and attack impact metrics [137] could be extended to consider different hierarchical operational layers. Though cost metrics can be hard to compute due to the difficulty of organizations in estimating security costs, one potential enhancement is to approach this category of metrics using high granularity estimation of costs to define acceptable thresholds [138,139].

In addition, considering the fact that 5G and/or IoT technologies are expected to affect current SIEM architectures due to the increased volume of data to be processed, it will be necessary to move towards a hierarchy of SIEMs and create collaborative mechanisms that will help notify and manage relevant security incidents. In the 5G domain, for instance, a SIEM solution is currently able to cover the analysis of one network slice; however, in the near future we will require collaboration mechanisms among multiple slices. Such a mechanism can be particularly useful in architectures where detection is required to be performed closer to the edge. In the IoT domain, for instance, having several SIEM systems working in different layers (e.g., SIEMs deployed in gateways) could be of great interest. These SIEMs must be lighter and more domain-specific than current solutions.

Furthermore, integration of SIEMs with extended detection and response (XDR) platforms is expected to provide value in two different but complementary ways: (i) having SIEMs focused on compliance and evolving to serve as a broader threat and operation risk platform, and (ii) having XDR focused on threats and providing a platform for deep and narrower threat detection and response. As a result, organizations would require solutions providing detailed level of information about the network and/or user activity taking place in the cloud or locally, to detect threats more accurately [140].

Finally, considering that the use of SIEMs generally require SOC operators and that current infrastructures are more diverse and dynamic, the next generation of SIEMs must focus on providing more autonomy and less effort in its deployment and management, which in turn will decrease their cost by simplifying their usage and operation.

## 7. SIEMs in Critical Infrastructures

Critical infrastructures (CIs) are organizational and physical structures whose failure and/or degradation could result in significant disruption of public safety and security. They rely on the Supervisory Control And Data Acquisition (SCADA) technology to monitor industrial and complex systems based on Networked Control Systems (NCSs). CIs include sectors that account for substantial portions of national income and employment (e.g., energy, water, transport, finance, health, etc.). Most of them use Industrial Control Systems (ICS) to provide control of remote equipment (using typically one communication channel per remote station) [141,142,143].

Security in computer networks must be distinguished from security in critical infrastructure networks, since the interactions among nodes in CI networks is done in real time at a physical level. A great effort has been dedicated on the usage and implementation of cybersecurity solutions in the protection of CI networks. Nevertheless, most of the current approaches used in the cyber domain are neither suited nor feasible to be implemented in the CI domain, making it a big challenge when it comes to protecting CIs against cybersecurity threats [144].

A key objective on protecting critical infrastructures is improving their security, which involves not only enhancing physical security, (e.g., ensuring physical rooms are locked appropriately to prevent access from unauthorized people), but also implementing effective cybersecurity measures to reduce the attack surface. Although a great effort has been made on the protection of CIs against cyber-attacks, they still present significant challenges e.g., it is not possible to execute a vulnerability scanning on an ICS as it is done in virtual systems since it may take the industrial system offline and thus, could bring down a plant’s operations [145].

While classical IT networks focus more on confidentiality and integrity (ensuring data is protected), ICS focuses more on availability (ensuring the system is always up and running). Industrial systems were not designed with security in mind, they were designed simply to be operative. They are generally legacy systems running on older operating systems, typically unpatched, and fragile in many cases. Although security strategies (e.g., network segmentation, firewalls, physical air-gaps, endpoint security, etc.) are deployed to decrease risk levels, they sometimes foster a false sense of security. Malicious entities can exploit gaps in corporate networks and move laterally into industrial systems to steal data or damage critical assets [146].

Security administrators require not only the collection of huge amounts of data, but also finding connections among these data in a way that can help identifying potential threats as well as defining appropriate mitigation strategies. Although this process has traditionally been performed through SIEM systems, current solutions are not able to fully detect all types of attacks affecting critical infrastructures [146]. In addition, considering the fact that attacks have increased both in number and complexity, organizations are obliged to improve their security by using tools with more advanced capabilities for the protection, detection, and reaction against cyber and physical attacks. SIEM systems are definitely an interesting solution to cope with these challenges. They are rapidly advancing into data analytic platforms that provide high-performance correlation functionalities and are able to raise alerts from a business perspective considering different alert aggregation methods [5] and events collected at different layers in real time [147,148,149].

The remainder of this section provides examples on the usage of SIEMs solutions in different industrial sectors.

### 7.1. Energy Distribution

The energy sector (including the production, storage, transportation, and refining of electrical power, gas, and oil) is particularly affected by cybersecurity threats and attacks. According to a recent study, three main aspects make of this sector vulnerable to cyber-attacks: (i) the increased number of threats and actors targeting utilities; (ii) the increased attack surface, arising from their geographic and organizational complexity; (iii) the unique interdependencies between physical and cyber infrastructure in the electric-power and gas sectors. As a result, energy companies are vulnerable to a wide range of threats including billing fraud with wireless “smart meters” and even physical destruction [150].

SIEMs are being considered as an essential solution to protect the energy industry against a variety of threat scenarios. A research study [151] performed on power grid infrastructures evaluated the benefits of SIEM solutions in detecting attacks (e.g., sleep deprivation, distributed denial of service, GPS spoofing). The SIEM technology used in this domain is able to perform techniques to monitor absolute and relative signal strengths and compare received ones against expected ones in order to identify anomalies in power grid infrastructures. As a result, an alarm is raised whenever a deviation is found, and valuable information is provided to the security analyst in order to mitigate and manage detected attacks. Thus, the use of SIEM technologies is proven to be beneficial in the protection of critical assets.

### 7.2. Water Supply

The water sector is also affected by cyber-attacks. Threat actors can attack water at its source, treatment plants, storage facilities, or distribution centers. SIEM solutions help monitor the entire SCADA network in real time to respond to any changes in the quality of water as soon as they are detected, as they might represent a potential attack. Current version of SIEMs such as the LogRhythm NextGen SIEM Platform [152] allow security administrators to effectively observe, collect, and analyze the data from the data historian in one interface, as well as identify any deviations from the acceptable ranges (e.g., for drinking water) during a specific period of time. Examples of attacks that can be detected by correlating security events in the industrial control network include reconnaissance, network behavior changes, changes in operator or engineering user behavior, detected or failed malwares, web-based attacks targeting human machine interfaces (HMI), man-in-the-middle attacks, etc.

One of the major challenges the water sector faces is the lack of cybersecurity situational awareness and the gaps in defense in depth mechanisms. The common belief in many sectors is that a high level of security can be achieved by deploying cutting edge technologies to protect and counter potential risks. However, defense in depth cannot be achieved if organizations do not clearly understand the relationship of vulnerabilities, threats and the mitigation measures used to protect the operations, personnel, and technologies of an ICS. Defense-in-depth is a holistic approach that considers the interconnections and dependencies among the aforementioned entities while protecting the organization’s assets and using their available resources to provide effective layers of monitoring and protection based on the business’s exposure to cybersecurity risks [153].

Next-generation SIEMs must enable multiple technologies to work together over IT and OT environments instead of operating in silos, so that organizations obtain automated responses to security incidents more quickly, have a complete visibility of their networks, and are able to plug OT security gaps as well as simplified management. Fortinet (https://www.fortinet.com/resources/cyberglossary/critical-infrastructure-protection accessed on 1 April 2021), is an example of such tools that offer protection for SCADA systems and ICS while enabling organizations to design the security of their infrastructures more efficiently and in compliance with current laws and regulations.

### 7.3. Transportation

Transport networks have become increasingly digitized, with a wide range of data flowing across systems, tracking and monitoring both digital and physical networks. As more devices and control systems are connected online, more vulnerabilities will appear, increasing the potential for disruption to physical assets. Threat actors can attack all transportation modes including aviation services, highway and motor carriers, maritime transport systems, and railway services [154].

As cyber technology becomes more sophisticated, the threat from attack is moving from data breaches to interrupting physical critical infrastructure, exposing transport operators to economic and reputational damage. Some of the key cyber risks affecting the transportation industry include physical asset damage and associated loss of use, unavailability of IT systems and networks, loss or deletion of data, data corruption or loss of data integrity, data breach, cyber espionage, extortion, theft, and damaged reputations. Most of these risks are realized through the exploitation of vulnerabilities that use social engineering techniques to deliver spam and phishing campaigns, inducing virus and malware installation (including ransomware) [155].

SIEM systems are essential in the improvement of the cyber and physical security in all transport services. Several solutions [156,157,158] have been proposed in the literature to protect various transport modes in the EU. As a result, it has been possible to develop cybersecurity plans aligned with the infrastructure’s overall strategy, to improve security in systems and applications, to have cybersecurity support for new developments, to raise employees’ awareness, to permanently manage security in both a preventative and reactive way, and to apply clearly defined security policies.

### 7.4. Healthcare

As medical procedures, diagnostics, and health data are becoming electronic, cloud-based, and distributed among numerous stakeholders, healthcare infrastructures have gained the attention of potential malicious third parties. According to an IBM survey [159], ransomware (or any kind of malware), social engineering (e.g., spear phishing), and bad practices adopted by staff and clients alike are the most common attack vectors in the sector.

In the near future, for the patient-centered healthcare model to fully function, sharing medical data and information between stakeholders and healthcare service providers is inevitable. The individualized patient approach, mobility, increased usage of personal medical devices, and commercial personal healthcare devices are making the roles of these devices and usage of their data even more indistinct. Technology and threats keep developing and only secure-by-design medical devices and services should be approved to healthcare networks. Nevertheless, there will be new cyber-attacks and new unknown vulnerabilities and threats, which is why the use of technologies (e.g., SIEM) is essential [160].

According to a recent study [161], the features that make SIEM solutions essential to be used by healthcare organizations are: (i) real-time analytics; (ii) self-learning configuration management database; (iii) scalabale log management; (iv) multi-tenant management; (v) compliance reports. SIEM solutions provide security administrators a consolidated and global look into organization’s security events which can prevent Health Insurance Portability and Accountability Act (HIPAA) violations and keep health data safe. While components of the healthcare infrastructure have their own security features, the ability to see all events in one dashboard is invaluable to protect data [162].

### 7.5. Financial Services

Financial organizations represent a major target for external and insider threats seeking financial gains or rewards. The major challenges faced by financial enterprises are three-fold: (i) business scaling, which exposes the sector to more potential attack vectors as the data is managed by third-parties through the use of clouds; (ii) legal and regulatory compliance, which restricts the use of personal identifiable data and requires the implementation of technologies according to privacy standards; (iii) insider threats (current or former), which generally go undetected and can cause serious harm to the business either out of ignorance or intentionally [163].

In terms of legal and regulatory compliance, a major challenge is related to a mandatory incident reporting to the competent and supervisor authorities and the need to compile information about incidents to generate and share reports that in many cases must be compliant with diverse regulations, procedures, templates, data sets, and other requirements. Although reporting is one of the key steps always present whenever a security incident takes place, there is not an agreement or a common procedure to be followed for incident reporting and sharing, even in the same sector such as the financial one. As a result, the lack of standards generates unstructured reports that cannot be easily analyzed. Key-search automated approaches for data extraction cannot be applied because they produce a high number of false associations in the analyzed reports. SIEMs must improve and simplify the process of collection and mandatory reporting and sharing of the information about major security incidents suffered by the financial institutions [85].

Modern SIEMs features User and Entity Behavioral Analysis (UEBA) to identify baseline behaviors of users, devices, and applications. Insider threats are therefore detected as soon as a user violates their baseline behaviors. In addition, SIEM solutions can detect data exfiltration through unusual network traffic and/or abnormal usage of internal resources by outsiders. SIEM solutions can also help financial enterprises achieve compliance through out-of-the-box reports and automatic report filling [164]. Other usages of SIEMs in the financial sector include account abuse (e.g., detect and respond to employees checking on dormant customer accounts), audit trial protection from unauthorized manipulation, forensics, and fraud detection [165].

## 8. Related Work

The analysis and evaluation of security systems have been widely proposed in the literature. While some research focuses on the commercial aspects, others concentrate on the technical features that could be improved in current SIEM solutions. Well known institutions like Gartner [20], for instance, propose a commercial analysis of SIEM systems based on the market and major vendors, for which a report is released on an annual basis to position SIEM vendors as market leaders, challengers, niche players, or visionaries. Although companies like Gartner periodically evaluate the capability of SIEMs, to the best of our knowledge, there is no systematic survey of these systems, their capabilities, and the open gaps.

In addition, other security institutions (e.g., Techtarget [166], Info-Tech Research Group [167]), have widely reported on the capabilities of SIEM solutions and on the way SIEM vendors can be compared and assessed. Techtarget, on the one hand, releases periodic electronic guides about securing SIEM systems and how to define SIEM strategy, management, and success in the enterprise [168]. Info-Tech, on the other hand, provides technical reports on the SIEM vendor landscape [169] focusing on the benefits and drawbacks of major commercial SIEMs. Both organizations take the Gartner Magic Quadrant as the baseline for their analysis, leaving aside the more technical aspects to be considered in future SIEMs.

Similarly, organizations such as Solutions Review [22] offer periodic reports to guide SIEM buyers on the appropriate selection of the SIEM solution for their businesses. Authors analyze key SIEM capabilities and perform a comparison vendor map based on three fundamental aspects (i.e., compliance, log management, and threat detection). Although the report allows connecting potential buyers with vendors, it does not provide technical details of the tools nor discusses about potential capabilities to be enhanced in current SIEMs, or external factors that could affect their performance in the future.

Caccia et al. [68], provide an analysis on the future of SIEMs by discussing aspects such as limitations of current SIEMs, the need for improvements in SIEM features, and the use of User and Entity Behavior Analytics (UEBA) for effective detection and efficient response. The authors focus on technical features to be enhanced in current SIEM solutions, but no details are given on the potential barriers and enablers to be considered in the development and implementation of future SIEMs.

Kotenko and Chechulin [170] propose a framework for attack modeling and security evaluation in SIEM systems applicable for future systems of the Internet of Things. The approach concentrates on technical features (e.g., evaluating the usage of comprehensive internal security repository, open security database, service dependency graphs, attack graphs, and security metrics) to be integrated into a SIEM framework in order to enhance its functionality. As a result, the authors claim to achieve more accurate and faster evaluations of network security aspects by the use of the proposed attack modeling and security evaluation component. Besides some technical aspects, no other features are considered for the improvements of current SIEM systems.

Based on the aforementioned limitations, we propose in this paper an analysis of current SIEM solutions based on commercial and technical features that could lead to enhancements in the design, development, and implementation of the next generation of SIEMs. The analysis focuses on the limitations of current SIEMs and on external factors that could potentially affect them in the long term. It includes a review and comparison of different commercial SIEMs during the last decade.

## 9. Conclusions

This paper presents a commercial and technical analysis of some of the leading SIEM solutions available in the market, namely ArcSight, QRadar, McAfee, LogRhythm, USM-OSSIM, RSA, Splunk, and SolarWinds. This choice has been based on the performance and trajectory of the companies developing this technology along the past decade.

In terms of behavioral analysis, and risk analysis and deployment, techniques and tools for analyzing, evaluating, and guiding the optimal deployment of diverse security mechanisms in the managed infrastructure (including multi-level risk-based metrics) must be developed along with a framework for deploying diverse and redundant sensors.

Although most of the analyzed solutions provide user-friendly graphical interfaces, visualization and reaction capabilities are limited to deal with huge numbers of collected events. It is therefore important to develop visualization and analysis extensions, which help give users a high-level of insight into the situation and a more efficient decision making and reaction capability.

With regards to data storage and price, although most of the solutions analyzed include good data storage capabilities, they are limited by the hardware availability and they usually require additional products (and licenses based on data volume) with a consequent increase in the price. Secure and elastic solutions based on cloud-of-clouds storage for long-term SIEM data archival in diverse public clouds (e.g., Amazon S3, Amazon Glacier, Windows Azure, Blob Store, etc.), are seen as promising alternatives with the ability to customize policies to fit data retention needs.

Finally, the role of the SIEMs has also been studied in the near and long-term future taking into account different aspects (e.g., political, economic, social, technological, environmental, and legal factors) in various critical infrastructures. From this analysis we can conclude that conditions are good to foster investment in improving and extending this technology as a key component not only for industrial control systems with security operation centers, but also to provide cyber security management for SMEs with reduced security knowledge and capacities.

## Figures and Tables

**Table 1:** SIEM vendors classification.

SIEM Vendor	2010	2011	2012	2013	2014	2015	2016	2017	2018	2020
HP/ArcSight/HPE [24]	★	★	★	★	★	★	★	⧫
RSA/EMC [25]	★	★	⧫	⧫	⧫	⧫	⧫	⧫	★	★
SenSage [26]	★	■	▲	▲
LogLogic [27]	★	★	⧫
Symantec [28]	★	★	⧫	⧫
Q1Labs [29]	★	★	★	★
Novell [30]	★	★	★
IBM [31]	⧫	⧫	★	★	★	★	★	★	★	★
Quest Software [32]	⧫	⧫
CA [33]	⧫
Tenable [34]	▲	■	▲	▲	▲
Prism Microsystems [35]	▲	■	▲
LogMatrix [36]	▲
NetIQ/Microfocus [37]	■	▲	★	⧫	⧫	▲	▲	▲	⧫	▲
McAfee/Intel [38]	■	★	★	★	★	★	★	★	★	▲
Trustwave [39]	■	■	■	▲	▲	▲	▲	▲
LogRhythm [40]	■	■	★	★	★	★	★	★	★	★
TriGeo [41]	■	■
netForensics [42]	■	■
eIQnetworks [43]	■	■	■	▲					
Splunk [44]		▲	⧫	★	★	★	★	★	★	★
Tripwire [45]		▲
AlienVault/ AT&T Cybersecurity [46]		▲	■	■	■	■	■	▲	▲	▲
Correlog [47]		▲	▲						
S21sec [48]		▲	▲						
Tango/04 [49]		▲	▲						
Tier-3 [50]		■	■						
SolarWinds [51]			■	⧫	▲	▲	▲	▲	▲	▲
Tibco-LogLogic [52]				■	⧫
EventTracker [53]				▲	▲	▲	▲	▲	▲
AccelOps/Fortinet [54]					▲	▲	▲	▲	▲	▲
Blackstratus [55]					▲	▲	▲	▲	▲
Manage Engine [56]							▲	▲	▲	▲
FireEye [57]								▲		▲
Venustech [58]								▲	▲
Rapid7 [59]								■	■	★
Exabeam [60]								■	★	★
Securonix [61]								■	★	★
LogPoint [62]									▲	■
HanSight [63]										▲

★ Leader ⧫ Challenger ▲ Niche Player ■ Visionary.

**Table 2:** SIEM tools/vendor characteristics.

ArcSight Enterprise Security Manager (MicroFocus/ HPE/ NetIQ)	Provides a graphical interface for the Security Operations Center (SOC) team and a set of applications or external commands that help the correlation and/or investigation processes.	Limited visualization options and intricate correlation rules [17]. The information associated with events is immutable, with evident deficits when it comes to adapting the product to company processes and needs.
Qradar (IBM)	Can be deployed as a hardware, software, or virtual appliance, as well as a Software as a Service (SaaS) on the IBM cloud. Provides a user interface for real-time event and view, reports, offenses, asset information, and product management. Offers support for threat intelligence feeds.	Provides basic reaction capabilities that include reporting and alerting functions. The endpoint monitoring for threat detection and response, or basic file integrity requires the use of third-party technologies.
McAfee Enterprise Security Manager (McAfee/ Intel)	Allows for scalable and versatile SIEM architecture, delivering real-time forensics, comprehensive application and database traffic/content monitoring, advanced rule and risk-based correlation for real-time as well as historical incident detection and automatic reaction.	Requires the use of additional solutions (e.g., McAfee Active Response). Predictive analytics and other built-in features such as behavioral analysis are poorly developed.
LogRhythm Next GEN SIEM Platform (LogRhythm)	Provides end-point monitoring, network forensics, user and entity behavior analytics, and response capabilities. Can be deployed in an appliance, software or virtual instance supporting scalable decentralized architectures	Unsuitable for organizations with critical infrastructures although extensions can be deployed to enhance the SIEM capabilities. Requires high degree automation and out-of-the-box content.
USM and OSSIM (AT&T Cybersecurity/ AlienVault)	Offers both commercial solutions (i.e., Alienvault Unified Security Management-USM ) and open source SIEM solutions. (i.e., OSSIM). Includes a web-based graphical interface for administration, reporting and security event management.	Limited user or entity behavior analytics as well as machine learning capabilities. Basic reaction capabilities (e.g., send email, execute script, open ticket) and limited to the pre-defined set of conditions associated to a security policy.
RSA Netwitness Platform (Dell)	Analyzes data and behavior of people and processes within a network across a company’s logs, packets, and end-points. Focuses on advanced threat detection. Provides strong OT monitoring capabilities	It requires a wide understanding of the breadth of the options and the implications for cost, functionality, and scalability.
Splunk Enterprise Security (Splunk)	Market-leading platform in Operational Intelligence. Offers data collection, indexing, and visualization capabilities for security events monitoring. Uses advanced security analytics, which include both unsupervised machine learning and user behavior capabilities.	Uses basic predefined correlation rules for monitoring and reporting requirements. Reaction capabilities are limited to email notifications. Requires integration with third-party applications for task and workflow automation.
SolarWinds Log and event Manager (SolarWinds)	Provides centralized log collection and normalization, automated threat detection and response, intuitive visualization, and user interface, as well as real time correlation and log searching to support investigation.	Lacks support for monitoring public cloud services’ IaaS or SaaS. Does not support custom report writing and customization of out-of-the-box compliance report templates.

**Table 3:** Analysis of different SIEM solutions.

Functionality	ArcSight	QRadar	McAfee	LogRhythm	USM-OSSIM	RSA	Splunk	SolarWinds
Correlation rules	∘	∘	•	•	•	∘	−	•
Data sources	•	•	•	∘	∘	•	•	∘
Real time processing	•	•	•	•	•	•	•	•
Data volume	•	∘	•	∘	∘	∘	•	∘
Visualization	−	∘	∘	∘	∘	∘	•	∘
Data analytics	∘	•	∘	•	∘	∘	•	∘
Performance	∘	∘	•	∘	∘	•	∘	•
Forensics	−	•	•	∘	•	•	∘	∘
Complexity	•	∘	∘	∘	∘	•	•	•
Scalability	•	•	•	•	−	•	•	•
Risk analysis	−	∘	∘	∘	−	∘	−	∘
Storage	∘	∘	•	∘	∘	∘	∘	•
Price	•	•	•	∘	∘	•	•	∘
Resilience	∘	•	•	∘	∘	•	∘	∘
Reaction and reporting	−	−	•	•	−	∘	∘	∘
UEBA	•	•	−	•	−	•	•	−
Security	•	•	−	−	∘	∘	∘	−

− Low/Basic ∘ Average • High/Advanced.

## Data Availability

Not applicable.
